# A Case Series on Uterine Arteriovenous Malformations: A Life-Threatening Emergency in Young Women

**DOI:** 10.7759/cureus.9410

**Published:** 2020-07-27

**Authors:** Rabia Hammad, Sidrah Nausheen, Mumtaz Malik

**Affiliations:** 1 Obstetrics and Gynaecology, Aga Khan University Hospital, Karachi, PAK; 2 Obstetrics and Gynecology, Aga Khan University Hospital, Karachi, PAK; 3 Radiology, Aga Khan University Hospital, Karachi, PAK

**Keywords:** arterio venous malformations, embolisation, irregular vaginal bleeding ultrasound

## Abstract

Uterine arteriovenous malformation (AVM) is a rare condition, with few cases reported in the literature. Despite being rare, it is a potentially life-threatening condition in women of child-bearing age. It should be considered in the differential diagnosis of prolonged or irregular vaginal bleeding, which, otherwise, can lead to critical complications ending up in severe morbidity and mortality. This case series describes four cases of young Asian women aged between 33 and 38 years who presented with irregular vaginal bleeding. Trans-abdominal ultrasound of the pelvis showed increased vascularity with multi-directional blood flow in the uterus. Magnetic resonance imaging (MRI) confirmed an arteriovenous malformation in all cases. All cases remained stable through the diagnostic journey. Embolization of the arteriovenous malformation was performed successfully in three cases and one case was managed conservatively on hormones. Later, two of them conceived within a year and had live births at term. The aim of reporting these cases is to share the common presentation of this condition and our experience in making the diagnosis and treatment of such patients. Although a few cases are reported world over, none was reported earlier from Pakistani Asian women.

## Introduction

Uterine arteriovenous malformation is a rare, life-threatening gynecological disease with less than 100 cases reported in the literature. It occurs as a result of abnormal and nonfunctional communication between the branches of uterine arteries and veins without an intervening capillary network [1-2]. Clinical presentations vary from irregular to heavy vaginal bleeding. It contributes to about 1%-2% of all genital and intraperitoneal hemorrhage and is usually diagnosed in women with unexplained vaginal bleeding at child-bearing age [3]. It may be congenital or acquired [4]. Congenital presentation is rare. Acquired uterine arteriovenous malformations occur mostly after damage to uterine tissue and are associated with conditions, such as pregnancy, uterine surgical procedure (cesarean section, curettage) [5-6], and, less commonly, cervical or endometrial carcinoma, infection, gestational trophoblastic disease, and exposure to diethylstilbestrol [7]. The diagnosis is easily made by using color Doppler ultrasonography showing a mosaic pattern and magnetic resonance imaging (MRI), which are non-invasive techniques. Pelvic angiography is also used for its confirmation. Hysteroscopy can also be used for diagnosis, but it is invasive [8]. The treatment of uterine arteriovenous malformation mainly depends on the patient’s age, symptoms, desire for fertility, and site of uterine arteriovenous malformation lesion. Treatment options include hysteroscopic resection of the lesion [8], laparoscopic coagulation of uterine vessels [9], selective embolization [10], and hysterectomy [11]. The conservative approach includes medical treatment with non-steroidal anti-inflammatory drugs (NSAIDs), methylergonovine maleate [12], danazol [13], and gonadotropin-releasing hormone (GnRH) agonist [14], but it is associated with a high failure rate and persistent bleeding in some cases.

This case series highlights our experience in Asian (Pakistani) women having this rare gynecological condition at our medical center and discloses a new differential diagnosis of irregular vaginal bleeding from this part of the world.

## Case presentation

Case 1

**Figure 1:**
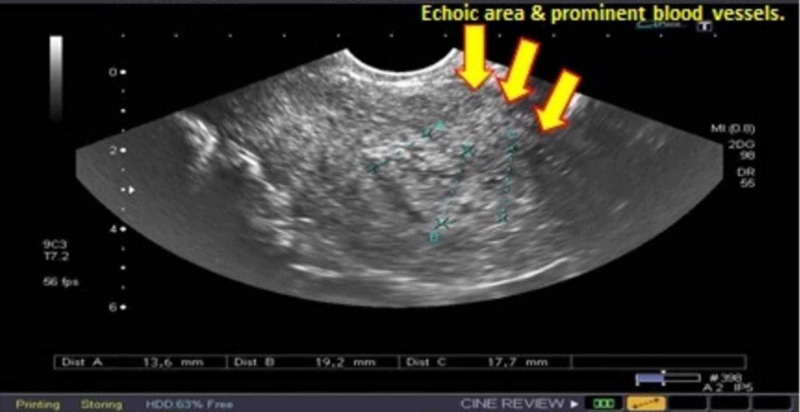
Pelvic ultrasound showing dilated blood vessels

A 32-year-old Asian woman, gravida 7 P3+2, with previous two scars, presented with seven weeks missed miscarriage. She underwent evacuation and was discharged in stable condition. She presented with heavy vaginal bleeding on the 10th postoperative day. There was no significant family history of gynecologic or genetic disease. Systemic examination was unremarkable except for pallor. Pelvic examination revealed bulky uterus, normal adnexa, and the os was closed with no active bleeding per vaginam. She was readmitted and the ultrasonography showed a bulky uterus with a complex mass measuring 7x6x11 cms in the lower part of the anterior wall of the uterus containing the multiple pools of blood and dilated vessels with blood flow (Figure 1). The histopathology report of products of conception revealed no trophoblastic disease. A provisional diagnosis of arteriovenous malformation was considered, which was later confirmed on MRI. Her hemoglobin was 6 gm/dl, and it was normochromic normocytic. Two pints of packed cells were transfused and selective uterine artery embolization was performed. During this procedure, abnormal tortuous vessels arising from branches of internal iliac arteries bilaterally and supplying the vascular malformation in the uterus were super-selectively cannulated using the microcatheter technique. Embolization was done using polyvinyl alcohol (PVA) particles of 335-1000 micron. Her bleeding stopped, and she was discharged home in stable condition. Later on, she menstruated normally. She was followed for 12 months during which she conceived spontaneously. Her pregnancy was low risk, with good maternal and fetal outcomes.

Case 2 

**Figure 2:**
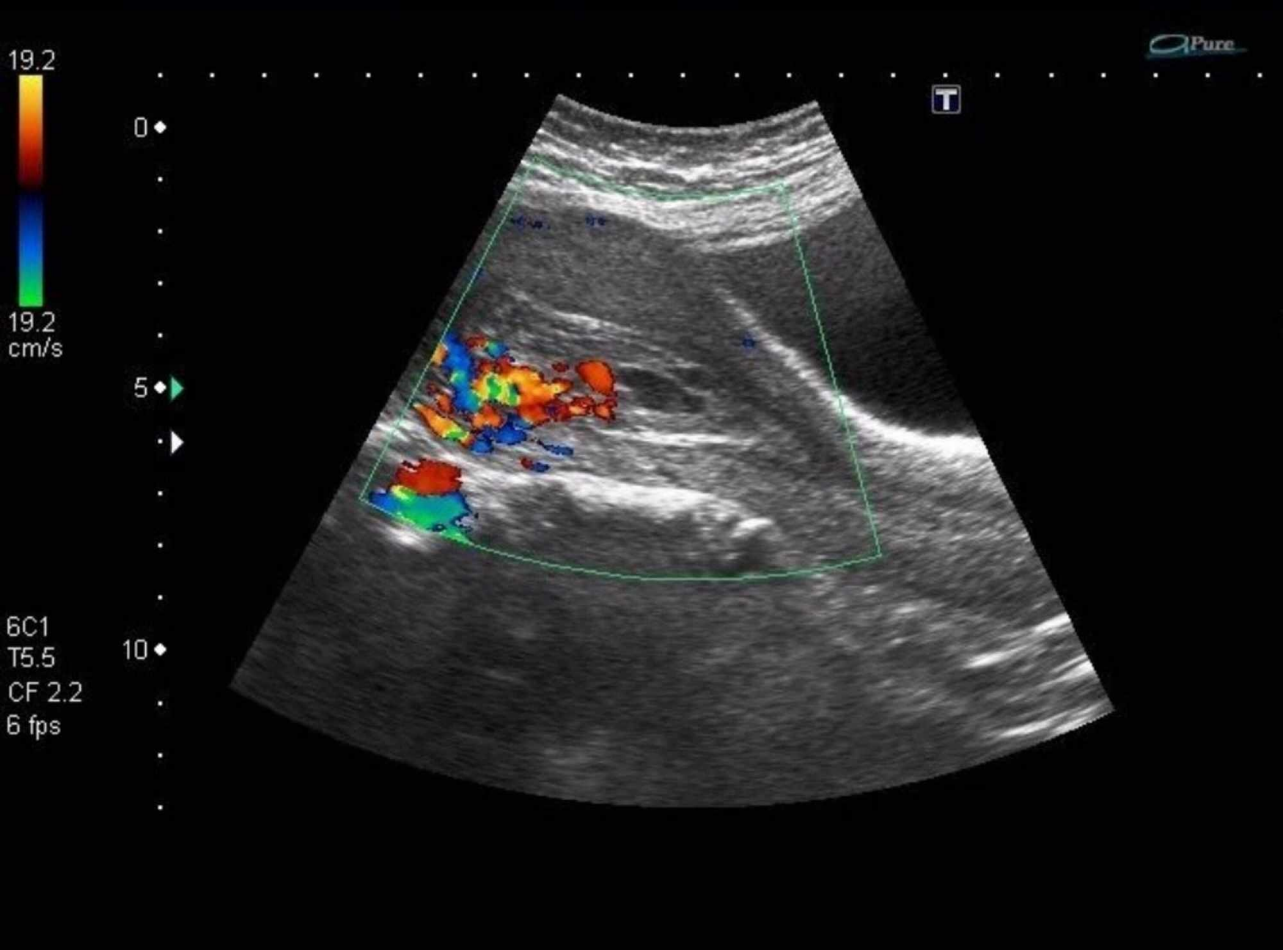
Doppler ultrasound showing hypervascularity in the endometrium

A 37-year-old Asian woman, para five, all born by spontaneous vaginal delivery and one first trimester miscarriage treated by dilatation and evacuation six months back, presented with eight weeks missed miscarriage. Dilatation and evacuation were performed without any complications. After 45 days, she reported in an emergency, with a single episode of profuse and heavy vaginal bleeding and a history of irregular vaginal bleeding for the last 40 days. She also took tab misoprostol 800 ug prescribed by a local practitioner in suspicion of retained products of conception but the bleeding continued. There was no past history of a bleeding disorder or a family history of any gynecologic cancer. On examination, she was in a state of hemodynamic shock, with cold clammy skin, blood pressure 90/40 mmHg, and a weak, low-volume pulse of 140/min. The chest was clear, with a respiratory rate of 20/min. Pelvic examination showed a bulky uterus with closed os and normal adnexa. On investigations, her hemoglobin was 5 gms/dl while platelets were normal. Red cell indices showed iron deficiency anemia. Electrolytes, clotting profile, and renal function tests were normal. The histopathology report of the previous evacuation revealed chorionic villi with no evidence of trophoblastic disease. Her beta-human chorionic gonadotropin (HCG) was less than 25 IU/L. She was resuscitated with two liters of intravenous (IV) normal saline. Bleeding was controlled with inj. tranexamic acid 1 gm stat and then eight hourly. Four pints of packed cells were transfused. Ultrasound Doppler was done, which showed a bulky uterus with irregular and thickened endometrium with some debris and blood clots. Vascularity on color flow suspected uterine arteriovenous malformation (Figure 2). She was planned for embolization but on refusal, was managed conservatively on oral progesterone 5 mg three times for 21 days. She remained asymptomatic for six months and had regular menstruation. Later, she opted for oral contraceptive pills as she wanted to defer pregnancy.

Case 3

**Figure 3:**
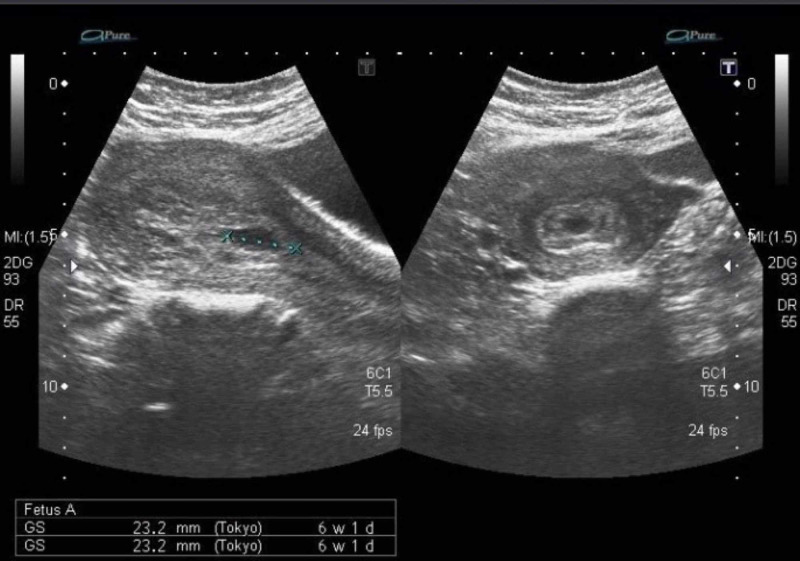
Pelvic ultrasound showing thick endometrium

A 35-year-old lady, gravida 4 para 2+1, previous vaginal deliveries, and last miscarriage followed by evacuation four months earlier was admitted with eight weeks missed miscarriage for the evacuation of the uterus. After one month of the evacuation, she presented to a local practitioner with heavy vaginal bleeding; evacuation was done again. The histopathology report showed no trophoblastic disease. After five days of this procedure, the patient reported to us in an emergency with heavy vaginal bleeding. On examination, she was hemodynamically stable with an unremarkable systemic examination. Her complete blood pictures and clotting profile was normal. Pelvic ultrasound was done, which showed a bulky uterus with mid-line thick irregular endometrium suspecting retained products of conception (Figure 3).

**Figure 4:**
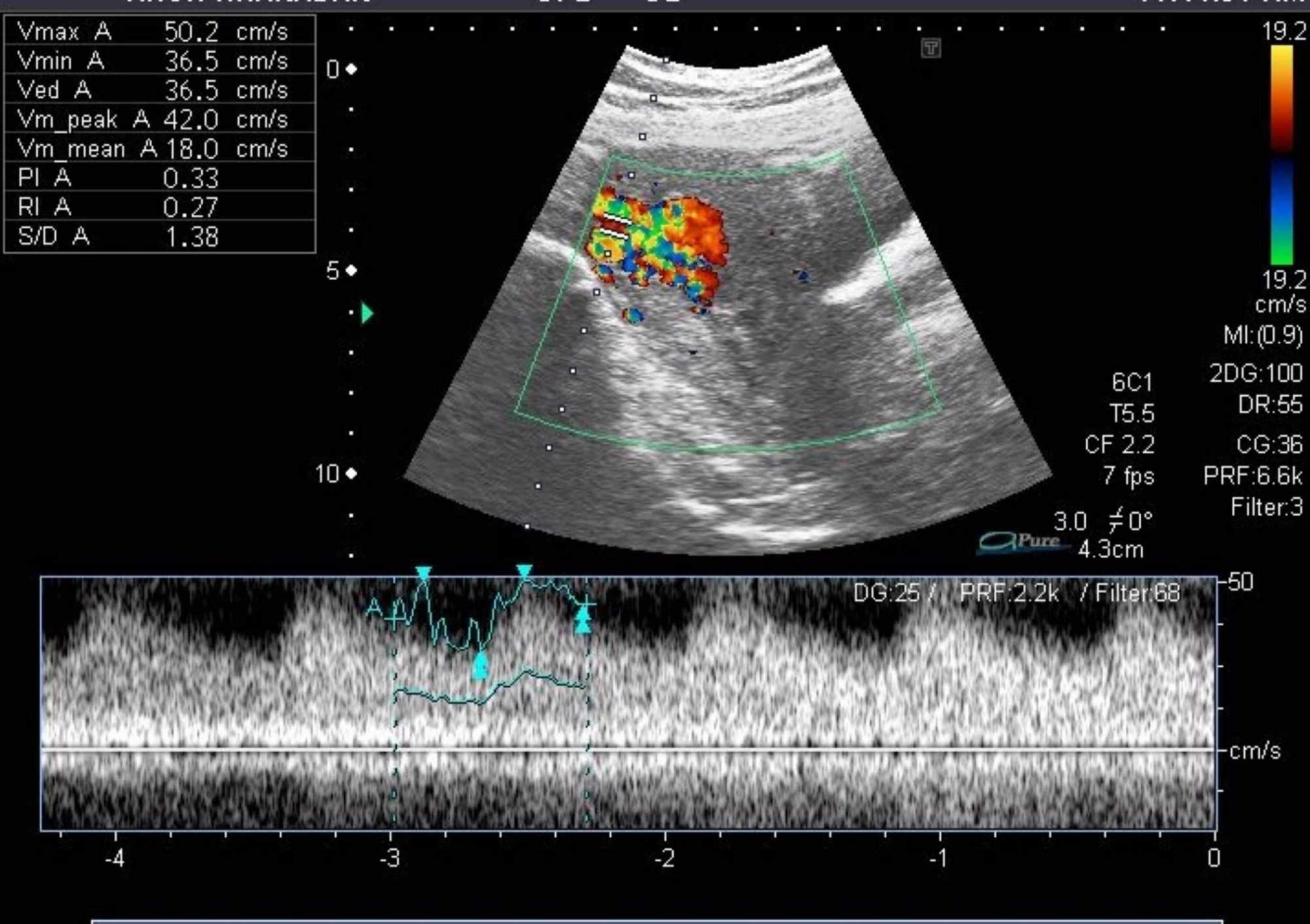
Doppler ultrasound showing increased vascularity in the endometrium

**Figure 5:**
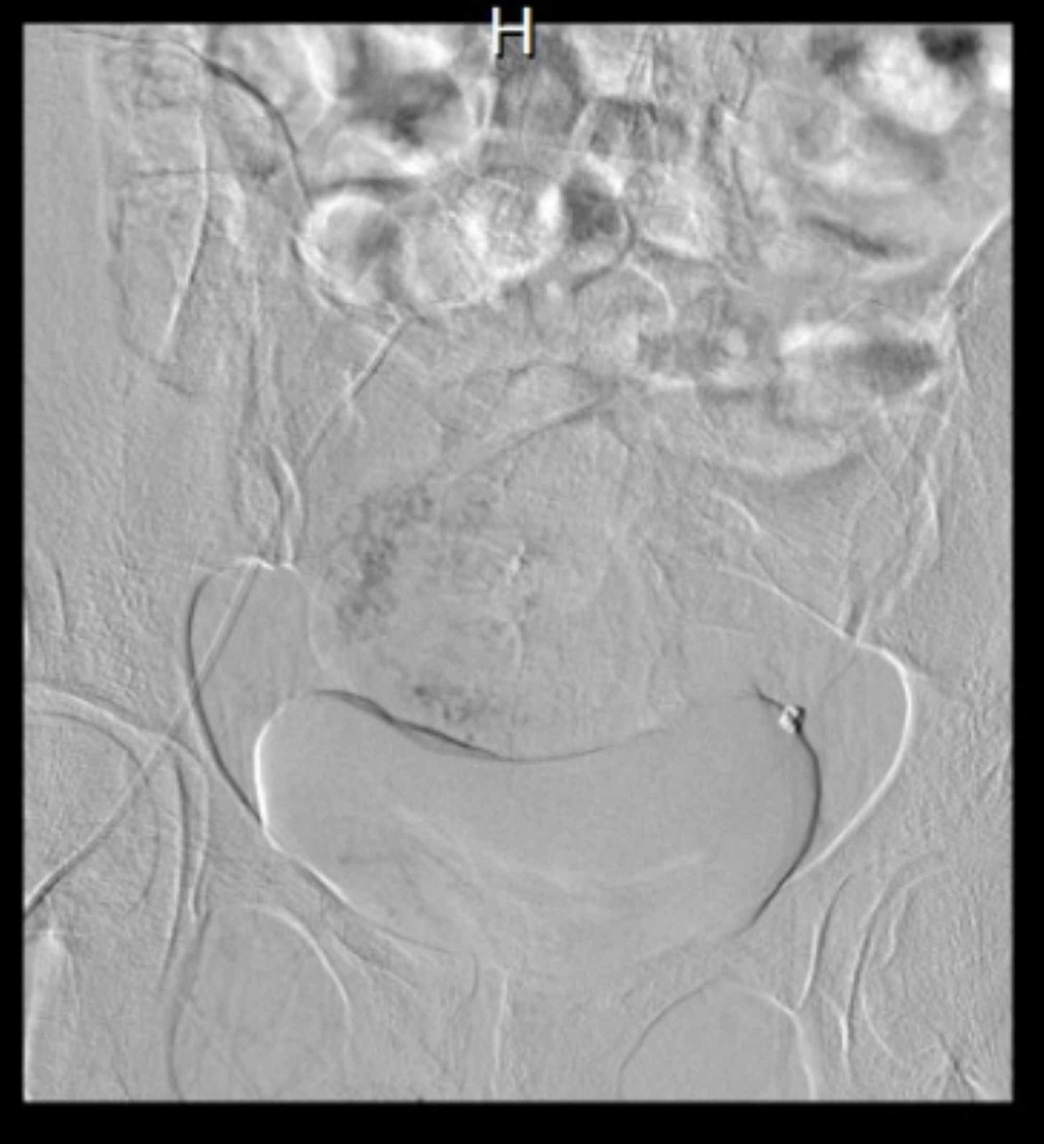
Selective uterine artery embolization

Color Doppler showed multiple blood vessels and a color mosaic pattern ( Figure 4). Her selective uterine artery embolization was subsequently performed (Figure 5). She was followed for 12 months, conceived spontaneously, had a low-risk pregnancy and delivered a healthy baby vaginally at term.

Case 4

**Figure 6:**
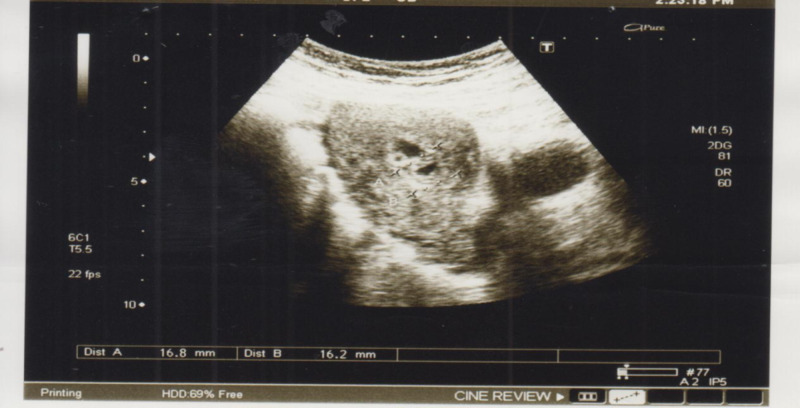
Pelvic ultrasound with thick endometrium and increased vascularity

A 38-year-old Asian woman, P5+1, all normal vaginal deliveries, presented with a history of dilatation and evacuation under general anesthesia for seven weeks and missed miscarriage two months back. Since the evacuation, she had multiple episodes of heavy vaginal bleeding, which resolved spontaneously without taking any medication. In between these episodes, she had mild irregular bleeding. On examination, she was afebrile and hemodynamically stable. Her hemoglobin level was 12.0 g/dL, HCG level was less than 2 mIU/mL, which ruled out trophoblastic disease. Pelvic examination revealed a normal-sized uterus and the os was closed with minimal bleeding. Transabdominal ultrasound of the pelvis showed a bulky uterus, with an endometrial thickness of 1.6 cm and increased vascularity of the uterus (Figure 6), which was later confirmed by Doppler ultrasound. A provisional diagnosis of arteriovenous malformation was considered, confirmed on MRI. Selective uterine artery embolization was done. Follow-up after 15 days, four weeks, and 12 months was uneventful. She was put on injectable progesterone as a method of contraception, as she did not desire pregnancy.

## Discussion

A uterine vascular abnormality is a rare but potentially life-threatening condition, which usually presents with vaginal bleeding that can be profuse and cause hemodynamic instability to the patient. It is considered an important differential in women of reproductive age with unexplained vaginal bleeding, as well as in post-menopausal women when the ultrasound reveals anechoic structures [15].

In 1926, the first case was reported by Dubreil and Loubart as a cricoid aneurism of the uterus and since then, it had been referred to as an arteriovenous aneurysm or arteriovenous malformations [16]. The true incidence is unknown, but with the increased use of ultrasound to evaluate abnormal vaginal bleeding, Obrien et al. proposed a rough, predicted incidence of 4.5% of all genital and intraperitoneal hemorrhages [3]. They can be congenital or acquired [2]. Congenital lesions are rare while acquired lesions are due to previous uterine surgeries, intrauterine devices, uterine infections, miscarriages, myomas, endometrial adenocarcinomas, endometriosis, and trophoblastic diseases.

Arteriovenous malformations consist of abnormal growths and connections between arteries and veins without a capillary bed, which are fragile and prone to bleed [17]. Congenital arteriovenous malformations occur due to an abnormality in the embryological development of primitive vascular structures, resulting in multiple abnormal communications between arteries and veins that invade the surrounding structures while the acquired results from trauma thus remain confined within the myometrium or endometrium, showing direct communication between the intramural branches of the uterine arteries and myometrial veins [18]. This is the basic difference between congenital and acquired forms.

The common presentation of arteriovenous malformation is usually in women of reproductive age, with a previous history of uterine surgery or procedures and vaginal bleeding, which may be intermittent or abundant, leading to hemodynamic instability as seen in three of our cases. Sometimes, pelvic pain is associated with vaginal bleeding [17]. Clinical examination can be unremarkable or with heavy vaginal bleeding [19]. Our reported case series presented the same classical pictures, i.e. women of reproductive age, a previous history of procedures, i.e. dilatation and evacuation with profuse vaginal bleeding, while one of them was in a state of shock.

An arteriovenous malformation is diagnosed initially with pelvic ultrasonography, in which it appears as a mass with multiple hypo/anechoic tubular structures of multiple sizes. Focal endometrial and myometrial thickenings can also be diagnostic. Doppler ultrasound adds the possibility of recognizing vessels within the malformations [18]. The confirmation is done with MRI or angiography. The typical MRI finding is a bulky uterus, with the presence of serpiginous and dilated vessels within the myometrium or parametrium [2,17]. Convention angiography, being the gold standard technique, shows enlarged, dilated irregular vascular spaces supplied by enlarged uterine arteries with high-flow dynamics [18]. Serum beta-HCG is recommended to exclude a gestational trophoblastic disease [18]. In our reported cases, first, a pelvis ultrasound was done, followed by Doppler, which further added to the possibility, and it was finally confirmed by MRI.

In the differential diagnosis, two conditions, i.e. retained products of conception and gestational trophoblastic disease, are important; they should be ruled out before final diagnosis. The gestational trophoblastic disease was ruled out in our four cases by confirming the histopathology report of retained products of conception and serum beta-HCG.

There is no clear consensus in the literature on the best treatment option for the disease. The current conservative medical or surgical options are based on the clinician’s expertise and a conclusion drawn from published case reports. The definitive treatment of arteriovenous malformation consists of surgical hysterectomy whereas conservative treatment is selective uterine arterial embolization [4,10,18]. The preferred treatment is selective uterine artery embolization in women of reproductive age who have a desire to conserve future fertility [4]. The definite surgical treatment, i.e. hysterectomy is reserved for those cases in which embolization is not feasible or contraindicated [2]. Three of our cases were managed by embolization successfully, with future fertility, as two of them had a live birth later on.

Another recently reported approach is the hysteroscopic resection of the uterine lesion by Stefano et al., from Italy [8], whereby 11 cases of arteriovenous malformations were managed successfully by operative hysteroscopy and four of them achieved a pregnancy that carried to term. The median duration of hysteroscopy was 30 minutes. This novel approach can be a breakthrough in the management of uterine arteriovenous malformations, as the author reported a 100% success rate with no surgical complications, high fertility outcomes, and short hospital stay. Further studies are required from other parts of the world to declare it as the standard treatment.

The conservative approach, i.e. medical treatment with combined oral contraceptive pills and progestogens is reported in some case reports for asymptomatic patients or with mild hemorrhage [20]. One of our cases was managed on hormones, although she was hemodynamically unstable, her bleeding stopped after injection tranexamic acid, and once she was stable, she was started on oral progesterone for 21 days and remained stable thereafter.

All patients remained stable through the diagnostic journey and had no morbidity and mortality. They were all appropriately diagnosed and managed. The prognosis after treatment was excellent and all patients were satisfied, which is one of the strengths. However, our limitation was the small sample size, as we report only four cases over a period of three years. We need more cases to see the diversity of the clinical presentation of the disease. The prognosis after treatment was excellent, and future fertility was conserved.

This case series highlights the importance of considering an arteriovenous malformation in a patient of childbearing age with heavy vaginal bleeding after a uterine procedure, the use of pelvic ultrasound, Doppler ultrasound, and MRI to confirm the diagnosis of arteriovenous malformation without any delay and to take a quick decision to perform selective uterine artery embolization to save her from life-threatening uterine bleeding and to retain her future fertility.

## Conclusions

Arteriovenous malformations are rare and dangerous clinical entities. Uterine bleeding, being one of the most common presenting complaints in women, can be life-threatening in specific situations. In a patient presenting with sudden and heavy vaginal bleeding, along with a history of prior uterine instrumentation, the possibility of uterine arteriovenous malformation should always be explored. Their management is complicated and challenging, requiring a high index of suspicion. Color or spectral Doppler ultrasonography are the best diagnostic tools that provide the most accurate information for confirmation. Hysteroscopy is an upcoming diagnostic and treatment modality. A clear understanding of the disease, a high index of suspicion, and the use of diagnostic modalities will bring a comprehensive clinical impact in the treatment of such difficult cases.
